# MR Angiography of Peripheral Arterial Stents: In Vitro Evaluation of 22 Different Stent Types

**DOI:** 10.1155/2011/478175

**Published:** 2010-07-27

**Authors:** Matthias C. Burg, Alexander C. Bunck, Harald Seifarth, Boris Buerke, Harald Kugel, Volker Hesselmann, Michael Köhler, Walter Heindel, David Maintz

**Affiliations:** Department of Clinical Radiology, University of Münster, Albert-Schweitzer-Str. 33, 48149 Münster, Germany

## Abstract

*Purpose*. To evaluate stent lumen visibility of a large sample of different peripheral arterial (iliac, renal, carotid) stents using magnetic resonance angiography in vitro. *Materials and Methods*. 21 different stents and one stentgraft (10 nitinol, 7 316L, 2 tantalum, 1 cobalt superalloy, 1 PET + cobalt superalloy, and 1 platinum alloy) were examined in a vessel phantom (vessel diameters ranging from 5 to 13 mm) filled with a solution of Gd-DTPA. Stents were imaged at 1.5 Tesla using a T1-weighted 3D spoiled gradient-echo sequence. Image analysis was performed measuring three categories: Signal intensity in the stent lumen, lumen visibility of the stented lumen, and homogeneity of the stented lumen. The results were classified using a 3-point scale (good, intermediate, and poor results). *Results*. 7 stents showed good MR lumen visibility (4x nitinol, 2x tantalum, and 1x cobalt superalloy). 9 stents showed intermediate results (5x nitinol, 2x 316L, 1x PET + cobalt superalloy, and 1x platinum alloy) and 6 stents showed poor results (1x nitinol, and 5x 316L). *Conclusion*. Stent lumen visibility varies depending on the stent material and type. Some products show good lumen visibility which may allow the detection of stenoses inside the lumen, while other products cause artifacts which prevent reliable evaluation of the stent lumen with this technique.

## 1. Introduction

Stenoses and occlusion of peripheral arterial vessels are frequently treated with angioplasty and stent insertions. After stent placement there is a risk of in-stent restenosis which can be caused by neointimal growth, vessel wall inflammation or stent thrombosis [1, 2]. For this reason, follow-up examinations are needed after successful revascularization therapy with stents. 

Different techniques are available for follow-up examinations. Intra-arterial digital subtraction angiography (DSA) has been the standard procedure for evaluating the stent patency for a long time. However, its invasiveness is afflicted with complications and a less-invasive alternative is preferable. Duplex sonography of arteries is completely noninvasive but it is operator-dependent and not commonly available for all body regions (e.g., renal or intracranial arteries) [3]. 

Spiral computed tomography angiography (CTA) is a noninvasive method to evaluate peripheral arteries and has been shown to be an alternative to intra-arterial DSA in different studies [4, 5]. Drawbacks of CT are the need of a potentially nephrotoxic iodine contrast material and ionizing radiation.

Three-dimensional (3D) contrast enhanced MR angiography (MRA) is another established noninvasive method to examine peripheral arteries. This method has been shown to be a promising noninvasive alternative to DSA and CTA for screening patients suspected of having lower extremity ischemia [6, 7]. However, MRA of stented arteries show difficulties due to imaging artifacts that may obscure the stent lumen [8–10]. Hamer et al. concluded that MRA is not yet a reliable technique to characterize in-stent restenosis because of high discrepancies between grading of lumen narrowing on DSA and MRA [11]. The magnitude of artifacts largely depends on the underlying stent type [12], and in the last several years new stent types appeared on the market that have not yet been evaluated for their MR lumen visibility.

Therefore, the purpose of this study was to evaluate the visibility and potential artifacts of different arterial stents in contrast to enhanced MRA in an experimental setting.

## 2. Material and Methods

### 2.1. Evaluated Stents and Experimental Setup

Twenty-one different stents and one stentgraft of different material and design were studied. Manufacturer, material, design, length, and nominal diameter of the stents and stentgraft are summarized in Table 1. Ten stents were made of nitinol, seven of stainless surgical steel (316 L), two of a cobalt-based alloy, two of tantalum, and one of a platinum alloy. The Wallgraft Endoprothesis is a Wallstent made of a cobalt-based alloy covered with polyethylene (PET).

The stents and the stentgraft were implanted into plastic tubes with 5, 7, 8, 10, or 13 mm inner diameter depending on their nominal diameter with one exception. The Palmaz Genesis Stent had a nominal diameter of 6 mm and was implanted into a 5 mm plastic tube. The wall of the small tubes (5, 7, and 8 mm) had a thickness of <0.3 mm whereas the wall of the large tubes (10 and 13 mm) was about 1 mm thick.

The tubes were introduced into plastic boxes which were filled with a solid gel (Dubliplast, Dentaurum AG, Pforzheim, Germany) which has a relaxivity similar to that of muscular tissue in the body [13]. The tubes were filled with gadopentetate dimeglumine (Magnevist, Schering AG, Berlin, Germany) in water at a concentration of 25 mmol/L (1 : 20 dilution of the standard preparation) and closed at both ends. All images were acquired on a 1.5 T MR system (Achieva 1.5 T, Philips, Best, The Netherlands). The phantom was positioned in the center of the magnet with an orientation parallel to the *z*-axis of the scanner. MR signal was obtained with a 5 element-phased array cardiac coil.

### 2.2. MR Imaging Parameters

We performed localizing sequences in three orthogonal planes. As MRA sequence we used a short 3D T1-weighted spoiled gradient-echo (T1-FFE) MRA sequence with the following parameters: TR = 3.4 ms, TE = 1.65 ms, Flip angle = 30°, field of view (FOV) = 290 × 200 × 50 mm, genuine voxel size = 1 × 1 × 1 mm, reconstructed voxel size = 0.55 × 0.55 × 0.55 mm, reconstruction matrix = 528 × 528, CLEAR (= **C**onstant **LE**vel **A**ppea**R**ance, Philips Medical Systems, Best, The Netherlands) = yes (for global image homogeneity correction based on coil sensitivity determination), and NSA = 2. Parallel acquisition techniques were not used. This sequence is a standard sequence clinically used for MRA of the peripheral vessels and not specifically adapted to improve stent visibility as proposed by previous studies [14, 15].

### 2.3. Image Analysis and Stent Evaluation

**Figure 1:**
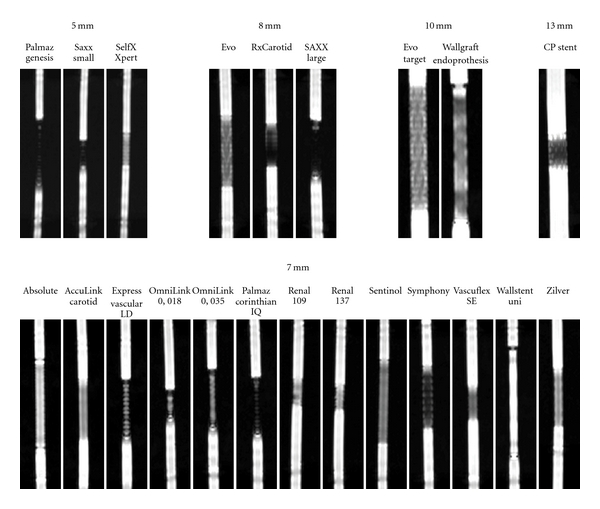
Comparison of 22 different peripheral arterial stents. Longitudinal MIP reformations are shown.

1. Image analysis was performed for all examined stents. Longitudinal maximum intensity projections (MIPs) were created for demonstration purposes only (Figure 1). For each stent, the middle slice intersecting the center of the stent parallel to its longitudinal axis was evaluated. Artifacts may obscure stent lumen in three different ways. The signal intensity may be decreased, the lumen may appear stenosed, and the signal intensity may be inhomogeneous. Therefore, every stent has been evaluated in the following categories: (1) Loss of signal intensity, (2) lumen narrowing, and (3) homogeneity of the lumen signal. For every category a grading was performed: good (score 3), intermediate (score 2), and poor (score 1) results.

For quantitative comparison of stent artifacts and lumen visibility, the loss of signal intensity was quantified by measuring the signal intensity inside the stented lumen and comparing it with the signal intensity in the unstented tube, calculating percent intensity. For the visualization of stents and for the possibility to detect stenoses a high-signal intensity inside the stent lumen is required. Therefore, we defined the following grading for the results of the signal intensity measurement: poor (1 point) <  40%, intermediate (2 points) 40%–60%, and good (3 Points) >  60%.

Furthermore, the visible lumen diameter inside the stented lumen was compared to the unstented vessel. To determine the lumen narrowing, we measured the maximal and the minimal diameter of the visible stent lumen. For our evaluation we focused on the minimal visible lumen diameter because if a stenosis is located at that position in the stent it can only be detected when there is even further loss of signal intensity than already caused by the static artifacts of a stent which is independent of lumen narrowing caused by a stenosis.

Regarding lumen narrowing, we defined the following grading: poor (1 point) <40%, intermediate (2 points) 40%–70%, and good (3 points) >70%.

The homogeneity of the signal intensity within the stented lumen was measured by defining a standardized region of interest (ROI) with a size of 120 pixels inside the stented lumen. We measured the standard deviation (SD) of the lumen signal and divided it by the mean intensity within the ROI to receive a numerical value of the homogeneity of the signal intensity. Based on these measurements, we defined the following grading: poor (1 point) ≥0.4, intermediate (2 points) <0.4 and ≥0.1, and good (3 points) <0.1. 

For the overall evaluation of the stent, the score of these three categories were summarized and resulted in an overall score between 3 and 9 with the following meaning: 3-4 poor, 5–7 intermediate, and 8-9 good.

## 3. Results

### 3.1. Signal Intensity

The signal intensities of the different stents are shown in Table 2. The relative signal intensity compared to the unstented tube ranged between 6% (Palmaz Genesis) and 117% (Wallstent Uni). 

After grading of the signal intensity, 6 stents were classified in the good group (3x nitinol, 2x  tantalum, 1x cobalt superalloy), 8 stents in the intermediate group (5x nitinol, 2x 316 L, 1x stentgraft of PET, and cobalt superalloy), and 8 stents in the poor group (2x nitinol, 5x  316 L, 1x platinum alloy).

### 3.2. Lumen Narrowing

The lumen visibility with respect to artificial lumen narrowing of the examined stents is shown in Table 2. 

In 4 stents we found a pattern of artifacts which results in a truncation of the signal of the stent lumen (Palmaz Genesis, Express Vascular LD, Palmaz Corinthian IQ and SAXX Large, all made from 316 L). The other stents showed visible lumen diameters between 27% (SAXX small) and 92% (Evo target). 

After grading the magnitude of artificial lumen narrowing for each stent, 7 stents were categorized in the good group (4x nitinol, 1x tantalum, 1x stentgraft PET + cobalt superalloy, 1x platinum alloy), 11 stents in the intermediate group (6x nitinol, 1x tantalum, 2x 316 L, 1x cobalt superalloy) and 5 stents in the poor group (5x 316 L).

### 3.3. Lumen Homogeneity

The lumen homogeneity of the stents is shown in Table 2. We classified 6 stents in the good group (4x nitinol, 1x cobalt superalloy, 1x tantalum), 10 stents in the intermediate group (5x nitinol, 1x tantalum, 2x 316 L, 1x PET + cobalt superalloy, 1x platinum alloy), and 6 stents in the poor group (1x nitinol, 5x 316 L).

### 3.4. Overall Lumen Visibility Results

The overall lumen visibility results are shown in Table 3. 7 stents exhibited overall a good lumen visibility in the contrast-enhanced MRA examination (4x nitinol, 2x tantalum, 1x cobalt superalloy): Absolute (nitinol), Renal 109 (tantalum), Renal 137 (tantalum), Sentinol (nitinol), Vascuflex SE (nitinol), Wallstent Uni (cobalt superalloy), and Zilver (nitinol). Furthermore, 9 stents showed intermediate results (5x nitinol, 2x 316 L, 1x PET + cobalt superalloy, 1x platinum alloy) and 6 stents showed poor results (1x nitinol, 5x 316 L).

## 4. Discussion

In the present study several different peripheral stents were examined in an MRI scanner with current electronics and gradient performance to evaluate their lumen visibility using magnetic resonance angiography (MRA) with a gadolinium-based contrast agent. 

The investigated stents are made from different materials (stainless steel (316 L), nitinol, cobalt superalloy, tantalum, and platinum-iridium alloy) and for different areas of application (arterial and peripheral arteries in general, biliary duct, carotid arteries, renal arteries, iliac arteries, femoral arteries, and the aorta). Independent from the area of insertion, follow-up examinations are necessary to determine the interventional success and exclude complications or restenosis. Contrast-enhanced MRA is one possible noninvasive modality for follow-up examinations and our study shows what to expect from this technique with a current MRI scanner and different stents.

A systematic explanation of the imaging features measured in this study is difficult. Signal intensity, signal homogeneity, and lumen narrowing are dependent on both, susceptibility and RF artifacts of the objects. The magnetic susceptibility difference between stent material and tissue causes local variations of the B_0_ field in the tissue adjacent to the stent material (which itself gives no signal). The field inhomogeneity results in misregistration of an acquired signal to a false position. In spin-echo images this causes bright spots adjacent to signal voids, while imaging with gradient echo techniques as in MR angiography causes additional signal loss in the area of local field inhomogeneities, resulting in larger signal voids, which may also mask the bright spots visible in spin-echo imaging [16, 17]. Shape and extent of susceptibility artifacts at a specific nominal field depend on the susceptibility of the stent material, the orientation of the stents relative to the B_0_ field, to the direction of frequency encoding (or phase encoding in the case of EPI), and to acquisition bandwidth.

With a fixed imaging protocol, larger susceptibility artifacts are to be expected for larger susceptibility differences between tissue and stent. For tantalum, nitinol, and platinum these differences are about 190 · 10^−6^, 255 · 10^−6^ and 290 · 10^−6^, respectively (field units in SI, [18]), for nonmagnetic steel 316 L in the order of 10^−3^ to 10^−2^. Differences of susceptibilities in different tissues (muscle, marrow, liver) are below 10 · 10^−6^ and in general negligible for imaging. Thus, for stents of equal geometry the size of artifacts increases in the order of tantalum, nitinol, platinum, steel, but this order may be changed depending on mass and configuration of the wire.

Another source of artifacts are radio frequency artifacts [19]. As stents are conductive structures, eddy currents through the struts of the stents occur during RF pulses, which cause a local shielding of B_1_, especially in the stent lumen. The actual B_1_ value strongly depends on the configuration of the wires in the stent. A local decrease of the B_1_ field results in a locally decreased excitation angle. In addition, the local B_1_ decrease is accompanied by decreased local signal uptake. A smaller pulse angle leads to an altered signal excitation; it can be increased, even higher than the signal outside the stent [15], if the image is T1 weighted and the lower pulse angle causes less saturation, that is, is nearer to the Ernst angle (see stent 15, Table 2). Otherwise the excitation is decreased. 

Therefore, as imaging features of stents are affected by various parameters of the material and configuration and a systematic prediction of artifacts of a specific stent is difficult, an individual assessment of stent imaging characteristics is necessary.

Using the modus of result evaluation with classification into three categories of stent visibility our study provides references on which stents are eligible for MRA examinations and which products should be examined with other techniques such as computed tomography angiography (CTA) or conventional intra-arterial digital subtraction angiography (DSA). In CTA, the magnitude of stent-related artifacts is correlated to the atomic number of the stent material (platinum (78), tantalum (73), nickel (28), cobalt (27), steel (26), and chromium (24)). It has been shown before [12] that stents which are not eligible for MRA examinations qualify good for CTA examinations and vice versa. Steel stents (316 L) show bad results in MRA and good results in CTA while tantalum stents show good results in MRA and bad results in CTA. 

With information about the imaging results of different peripheral stents in different examination techniques, the clinical radiologist can decide which technique is suitable for a follow-up examination of his patient.

The phantom used was designed to simulate conditions comparable to an in vivo MRI examination. Nevertheless, some limitations have to be considered. In our scans the stents were positioned parallel to the *z*-axis of the scanner. This would fit to the position of an aortic stent but not to renal or iliac stents. In other studies it has been shown that the artifacts caused by the stents depend on the angle between stent and the *z*-axis of the magnetic field [8, 14]. 

We used a static fluid model without flow within the stents. The effect on the characteristics of the artifact expression is assumed to be negligible because we used an MRA protocol with a gadolinium-based contrast agent, which measures the T1 differences of the materials and does not depend on the flow, in contrast to MRA examinations without contrast enhancement as Time of Flight or Phase Contrast techniques. 

Software algorithms like CLEAR for data postprocessing have been released to further improve image quality and reduce contrast inhomogeneities caused by parallel image acquisition. Thus, these data modification algorithms may have influence on SI measurements and therefore hamper SNR/CNR calculation. 

However, a recent study confirmed, that SI and SD measurements performed within the examined object are applicable in data sets reconstructed with a CLEAR-based algorithm and SNR/CNR calculations are valid [20]. 

We performed our scans in a 1.5 T MRI scanner. Other studies [21, 22] have shown that similar results can be expected from scans in a 3.0 T MRI scanner. There might be a higher signal-to-noise ratio and larger area of signal void surrounding the stents at 3.0 T compared to 1.5 T, but after all it seems likely that good stents at 1.5 T MRA will still belong to the better ones at 3.0 T MRA, and bad stents at 1.5 T MRA will also be worse at 3.0 T.

Our catalogue of the examined stents is not complete. Some of the currently available stents have not been included in this study, others have been investigated in previous studies [10, 12, 21]. Nevertheless, we examined stents of different materials, sizes, and designs so that our results can be transferred to similar products on the market. 

In summary, tantalum stents seem to be most suitable for MRA examinations. Stents made from a cobalt alloy or nitinol are still suitable for MRA examinations but they cause more artifacts than tantalum stents and it depends on their individual stent design if small stenoses can be detected. Stents made from stainless steel (316 L) show the worst results in our study though some of them still allow to detect relevant stenoses. 

## Figures and Tables

**Table 1:** Name, manufacturer, material, primary area of use, diameter, and length of the examined stents.

No.	Name	Manufacturer	Material	Primary area of use	Diameter (mm)	Length (mm)
1	SAXX Small	Devon	316 L	Arterial vessels	5	17
2	SelfX Xpert	Abbott	nitinol	Peripheral vasculature/biliary duct	5	20
3	Palmaz Genesis	Cordis	316 L	Peripheral arteries below aortic arch/biliary tree	6	39
4	Absolute	Guidant	nitinol	Biliary/peripheral vessels	7	60
5	AccuLink Carotid	Guidant	nitinol	Internal carotid and common carotid arteries	7	40
6	Express Vascular LD	Boston Scientific	316 L	Peripheral vessels	7	37
7	OmniLink 0,018	Guidant	316 L	Biliary/peripheral arteries	7	18
8	OmniLink 0,035	Guidant	316 L	Biliary/peripheral arteries	7	35
9	Palmaz Corinthian IQ	Cordis	316 L	Peripheral vessels	7	40
10	Renal109	Abbott	tantalum	Renal artery	7	18
11	Renal137	Abbott	tantalum	Renal artery	7	18
12	Sentinol	Boston Scientific	nitinol	Peripheral vessels	7	59
13	Symphony	Boston Scientific	nitinol	Iliac artery	7	40
14	Vascuflex SE	B. Braun	nitinol	Peripheral vessels	7	20
15	Wallstent Uni	Boston Scientific	cobalt-superalloy	Iliac artery, sfa (superficial femoral artery), tracheal	7	60
16	Zilver	Cook	nitinol	Carotid artery	7	40
17	Evo	pfm	nitinol	Pelvic arteries and peripheral vessels/biliary	8	50
18	RxCarotid	Abbott	nitinol	Carotid artery	8	30
19	SAXX Large	Devon	316 L	Arterial vessels	8	35
20	Evo Target	pfm	nitinol	Intravascular/biliary	10	80
21	Wallgraft Endoprothesis	Boston Scientific	braided polyester graft bonded to the outside of a Wallstent (cobalt-superalloy)	Trachea/bronchus (off label: peripheral arteries)	10	70
22	CP Stent	pfm	90% platinum 10% iridium	Aorta	13	28

**Table 2:** Results of signal intensity measurements, visible lumen measurements, and lumen homogeneity measurements.

No.	Name	% of signal intensity inside the stented lumen	Score signal intensity	Visible lumen diameter (%)	Score lumen narrowing	SD Lumen/Mean Lumen	Score homogeneity
1	SAXX Small	7	1	27	1	0.43	1
2	SelfX Xpert	62	3	58	2	0.11	2
3	Palmaz Genesis	6	1	0	1	0.44	1
4	Absolute	80	3	69	2	0.06	3
5	AccuLink Carotid	57	2	69	2	0.10	2
6	Express Vascular LD	21	1	0	1	0.48	1
7	OmniLink 0,018	46	2	38	2	0.33	2
8	OmniLink 0,035	45	2	51	2	0.21	2
9	Palmaz Corinthian IQ	12	1	0	1	0.42	1
10	Renal109	66	3	72	3	0.16	2
11	Renal137	69	3	58	2	0.07	3
12	Sentinol	51	2	89	3	0.04	3
13	Symphony	15	1	60	2	0.35	2
14	Vascuflex SE	42	2	80	3	0.09	3
15	Wallstent Uni	117	3	60	2	0.08	3
16	Zilver	66	3	66	2	0.07	3
17	Evo	45	2	80	3	0.19	2
18	RxCarotid	10	1	65	2	0.81	1
19	SAXX Large	7	1	0	1	0.40	1
20	Evo Target	43	2	92	3	0.16	2
21	Wallgraft Endoprothesis	52	2	75	3	0.16	2
22	CP Stent	21	1	86	3	0.14	2

**Table 3:** Overall results.

No.	Name	Score signal intensity	Score lumen narrowing	Score lumen homogeneity	Overall Score	Overall Evaluation
1	SAXX Small	1	1	1	3	Poor
2	SelfX Xpert	3	2	2	7	Intermediate
3	Palmaz Genesis	1	1	1	3	Poor
4	Absolute	3	2	3	8	Good
5	AccuLink Carotid	2	2	2	6	Intermediate
6	Express Vascular LD	1	1	1	3	Poor
7	OmniLink 0.018	2	2	2	6	Intermediate
8	OmniLink 0.035	2	2	2	6	Intermediate
9	Palmaz Corinthian IQ	1	1	1	3	Poor
10	Renal109	3	3	2	7	Good
11	Renal137	3	2	3	8	Good
12	Sentinol	2	3	3	8	Good
13	Symphony	1	2	2	5	Intermediate
14	Vascuflex SE	2	3	3	8	Good
15	Wallstent Uni	3	2	3	8	Good
16	Zilver	3	2	3	8	Good
17	Evo	2	3	2	7	Intermediate
18	RxCarotid	1	2	1	4	Poor
19	SAXX Large	1	1	1	3	Poor
20	Evo Target	2	3	2	7	Intermediate
21	Wallgraft Endoprothesis	2	3	2	7	Intermediate
22	CP Stent	1	3	2	6	Intermediate
